# Partial sequencing analysis of the *NS5B* region confirmed the predominance of hepatitis C virus genotype 1 infection in Jeddah, Saudi Arabia

**DOI:** 10.1371/journal.pone.0178225

**Published:** 2017-05-26

**Authors:** Sahar EL Hadad, Hesa Al-Hamdan, Sabah Linjawi

**Affiliations:** 1 Department of Biological Science, Faculty of Science, King Abdulaziz University, Jeddah, Saudi Arabia; 2 Research Center of Genetic Engineering and Bioinformatics, VACSERA, Cairo, Egypt; Centers for Disease Control and Prevention, UNITED STATES

## Abstract

Chronic hepatitis C virus (HCV) infection and its progression are major health problems that many countries including Saudi Arabia are facing. Determination of HCV genotypes and subgenotypes is critical for epidemiological and clinical analysis and aids in the determination of the ideal treatment strategy that needs to be followed and the expected therapy response. Although HCV infection has been identified as the second most predominant type of hepatitis in Saudi Arabia, little is known about the molecular epidemiology and genetic variability of HCV circulating in the Jeddah province of Saudi Arabia. The aim of this study was to determine the dominance of various HCV genotypes and subgenotypes circulating in Jeddah using partial sequencing of the *NS5B* region. To the best of our knowledge, this is the first study of its kind in Saudi Arabia. To characterize HCV genotypes and subgenotypes, serum samples from 56 patients with chronic HCV infection were collected and subjected to partial *NS5B* gene amplification and sequence analysis. Phylogenetic analysis of the *NS5B* partial sequences revealed that HCV/1 was the predominant genotype (73%), followed by HCV/4 (24.49%) and HCV/3 (2.04%). Moreover, pairwise analysis also confirmed these results based on the average specific nucleotide distance identity: ±0.112, ±0.112, and ±0.179 for HCV/1, HCV/4, and HCV/3, respectively, without any interference between genotypes. Notably, the phylogenetic tree of the HCV/1 subgenotypes revealed that all the isolates (100%) from the present study belonged to the HCV/1a subgenotype. Our findings also revealed similarities in the nucleotide sequences between HCV circulating in Saudi Arabia and those circulating in countries such as Morocco, Egypt, Canada, India, Pakistan, and France. These results indicated that determination of HCV genotypes and subgenotypes based on partial sequence analysis of the *NS5B* region is accurate and reliable for HCV subtype determination.

## Introduction

**Fig 1:**
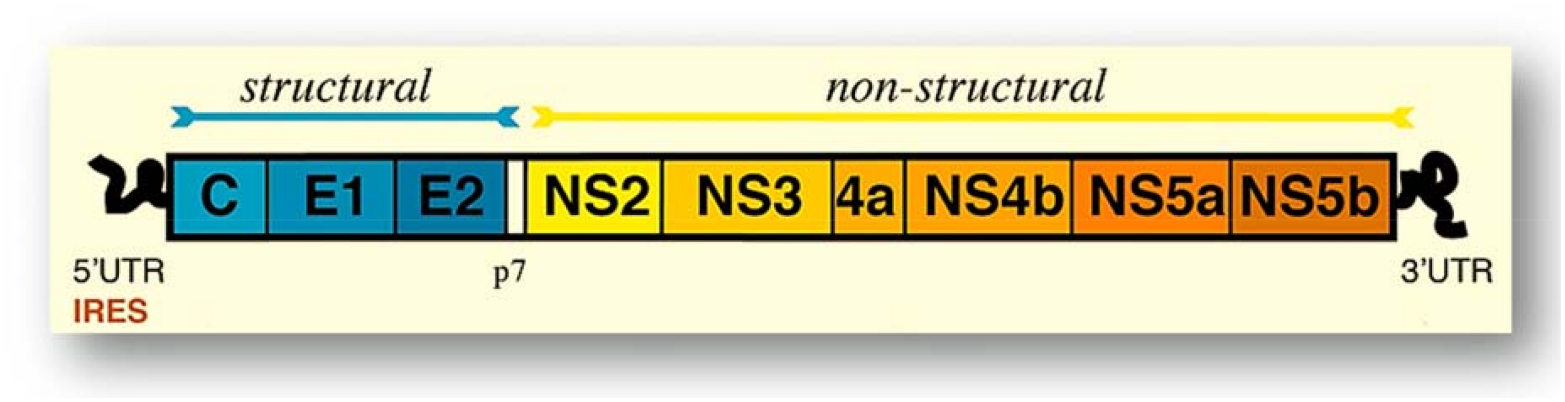
HCV genome organization. The HCV genome consists of an open reading frame (ORF) of approximately 9400 bp, which is translated as a single polypeptide of approximately 3000 amino acids. The polypeptide is cleaved to produce 10 mature protein products, and the positions of the hypervariable regions 1 and 2 are shown [8].

Hepatitis C virus (HCV) is an important human pathogen, which is estimated to infect 130–170 million people worldwide. HCV infection leads to chronic hepatitis (CHC), which in turn leads to liver steatosis, fibrosis, liver cirrhosis (LC), and hepatocellular carcinoma (HCC) [1–2]. HCV is a member of the *Hepacivirus* genus of the *Flaviviridae* family of viruses. The HCV genome is small and enveloped with one single-stranded positive-sense RNA molecule of approximately 9600 bp, structured in a coding region that contains one large open reading frame (ORF) flanked by non-translated regions (NTR) at the 5’ and 3’ ends, which encodes a polyprotein precursor of about 3,000 amino acids. The precursor is cleaved into at least 10 different proteins comprising the structural proteins, core, E1, E2, and p7, as well as the non-structural proteins, NS2, NS3, NS4A, NS4B, NS5A, and NS5B (Fig 1) [3–7].

Previously, HCV was classified into six major genotypes [9]; however, recently, seven major genotypes and numerous subtypes have been reported [10]. HCV genotypes 1 and 2 are primarily predominant in West Africa, 3 in South Asia, 4 in Central Africa and the Middle East, 5 in Southern Africa, and 6 in South East Asia [11–15]. To date, only one infection caused by HCV genotype 7 has been reported, where the virus was isolated in Canada from a Central African immigrant [16]. HCV infection has been defined as a reportable disease in Saudi Arabia since 1990 [17]. However, the prevalence of HCV in Saudi Arabia varies between different provinces; according to a report by the Saudi Government produced upon monitoring HCV incidence among 13 Saudi Arabian administrative provinces from 1995 to 2006, the highest prevalence is observed in western provinces such as Al-Baha and Jeddah (0.32%), and the lowest prevalence is in southern provinces such as Jizan (0.016%) [18]. Descriptions of HCV epidemiology in the Kingdom of Saudi Arabia (KSA) have led to HCV infection being considered a major public health problem in the KSA, especially among hemodialysis patients and intravenous drug users (IDU) [19–21]. A total of 437,292 official reports of HCV infections among persons living in the KSA have been submitted to the WHO, which gives an estimated prevalence of about 1.8% overall [22–23]. Several previous studies conducted in Saudi Arabia have proven that HCV genotypes 4, 1 [24–26], and 2 are the most prevalent genotypes, followed by genotype 3.

Definition of the HCV genotype is now a part of the pre-treatment process for patient management. It is also useful for inspecting outbreaks of infections and for understanding the epidemiology and biological features of this virus. Several methods targeting different regions of the HCV genome have been used for assessing HCV genotypes. The most accurate method so far is to sequence an appropriate coding region that is divergent enough to allow the discrimination of the genotypes and subgenotypes [27–28]. Many genotyping methods targeting different regions of the HCV genome, such as 5'NTR, the core (C), Envelope (E1), and 5’ noncoding region (*NS5B*) have been used in previous studies [9].

Since the 5'NTR is one of the most highly conserved regions of the HCV genome, it has historically been used for virus detection and is now one of the best-characterized regions. For practical reasons, the 5'NTR has also been chosen as the target for various genotyping methods including the InnoLipa HCV II test [29–30], sequencing [31], and the duplex mobility assay [32]. In this study, we analyzed the sequence of the *NS5B* region to determine the HCV genotypes and subgenotypes of 49 isolates collected from chronic HCV patients in the Jeddah province between 2014 and 2015. All the samples had already been genotyped based on 5’NTR analysis.

The current study was performed to determine the genotypes and subgenotypes using phylogenetic analyses of HCV strains collected from chronically infected patients in Saudi Arabia to establish a simple, accurate, and a reliable genotyping system for HCV for use in diagnosis. To the best of our knowledge, this is the first study determining HCV subtypes based on analysis of the nucleotide sequence of the *NS5B* region in the Jeddah province of Saudi Arabia.

## Materials and methods

### Patient samples

Serum samples were collected from 56 patients with chronic HCV infection from Saudi Arabia; S1 Table contains the data for all the patients. The patients were randomly selected based on availability from different hospitals in Jeddah; a review of the medical records confirmed that all the samples were positive for anti-HCV antibodies and that none of the patients had co-infection with HIV or HBV. The samples were divided into aliquots and stored at −70°C until use.

The study protocol was reviewed and approved by the Deanship of Scientific Research Ethical Committee of King Abdulaziz University and the King Abdulaziz Hospital Ethical Committee. Written informed consent was obtained from all the patients after full explanation of the purpose of the study.

### HCV-RNA extraction and HCV-NS5B amplification

HCV RNA was obtained from all the serum samples using the Mini Elute Viral Extraction Kit (QIAGEN, Inc., Valencia, CA, USA) according to the manufacturer’s instructions. The resultant HCV RNA samples were stored at −70°C until use [33]. The presence of the *HCV-NS5B* gene was determined by nested PCR using the One-step RT-PCR Master Mix kit and the Hot start *Taq* plus PCR Master Mix Kit (QIAGEN) using the primers shown in Table 1. The first round of RT-PCR amplification was performed according to the manufacturer’s instructions using 10 μL HCV-RNA and 50 *pmol* each of primers NS5BOS1 and NS5BOAS2. The cycling conditions were as follows: a reverse transcription step for 30 min at 50°C, 15 min at 95°C, followed by 35 cycles of denaturing for 1 min at 95°C, annealing for 45 s at 59°C, and an elongation step for 1 min at 72°C, with a final extension period of 10 min at 72°C. Nested PCR using the NS5BIS1 and NS5BIAS2 primers was performed on 10 μL of the samples negative for PCR products in the first round of amplification. The second round of amplification was performed with an initial 5 min preheating step at 95°C, followed by 35 cycles of denaturing for 30 s at 95°C, annealing for 30 s at 55°C, and elongation for 1 min at 72°C, with a final extension period of 10 min at 72°C. All the PCR contamination precautions were taken to ensure specificity of the reaction [16, 34–35].

**Table 1:** Primers used for partial HCV-NS5B amplification and their nucleotide positions.

Primer Code	Primer Sequence	Primer Length	Nucleotide Position
**NS5BOS1**	5'-TGGGGTTCTCGTATGATACCC-3'	21	8337–8357
**NS5BOAS2**	5'-CCTGGTCATAGCCTCCGTGAA-3'	21	8729–8708
**NS5BIS1**	5'-GATACCCGCTGCTTTGACTC-3'	20	8350–8370
**NS5BIAS2**	5'-CCTCCGTGAAGGCTCTCAG-3'	19	8717–8698

Nucleotide position numbering is based on the HCV reference sequence contained in ~9600 bp, which was retrieved from DDBJ/EMBL/GenBank (Accession number: JF735132).

### Interpretation of HCV-NS5B partial nucleotide sequences

Purified nested PCR products were sequenced in both directions using a Big Dye Terminator v3.1 Cycle Sequencing kit (Applied Biosystems (ABI), Foster City, CA, USA). The ABI Prism 310 genetic analyzer was used for electrophoresis and data collection per the manufacturer's protocol. All the sequences were assembled using SeqMan II software (DNAStar Inc., Madison, WI, USA) and multiple alignments with the reference sequences of *HCV-NS5B* genotypes and subgenotypes (1–6) were confirmed using CLUSTALW software [36].

### Phylogenetic analysis

Phylogenetic analysis of the amplified regions from each sample, corresponding to the partial *NS5B* gene, was performed to analyze the nucleotide heterogeneity of the isolates with the reference HCV genotypes/subtypes retrieved from the DDBJ/EMBL/GenBank databases (S2 Table). Phylogenetic trees were constructed by MEGA 6 software using the neighbor-joining method based on the Tamura-Nei model of evolutionary distance, and the topology was evaluated by bootstrap analysis (1,000 replicates) [36–40].

### Nucleotide sequence submission

The base sequence data as well as partial sequence data reported in this study have been submitted to DDBJ/EMBL/GenBank under the accession numbers KX774463, (KX810070-KX810071), (KX810077-KX810086) and (KX784096-KX78129) (S3 Table).

## Results

### Amplification of the HCV-NS5B region using nested PCR

**Fig 2:**
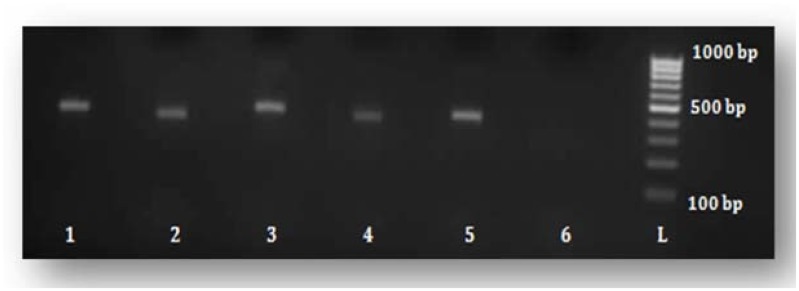
Amplification of the *NS5B* gene using nested PCR. “L” represents the 100-bp DNA ladder ranging from 100–1000 bp. Lanes 1, 2, 3, 4, and 5 contain DNA fragments of the *HCV-NS5B* gene, whereas lane 6 shows negative results.

Nested RT-PCR confirmed the presence of the *HCV-NS5B* gene in 49 (87.5%) of the 56 anti-HCV positive samples. The remaining seven (12.5%) samples did not show the presence of *HCV-NS5B* even after the second round of PCR amplification, which may have been due to low viral load. All the positive PCR products were of the expected size of approximately 400–500 bp, which included almost the partial *NS5B* genomic region (Fig 2).

### Identification of HCV genotypes based on the partial nucleotide sequence of the NS5B gene

**Fig 3:**
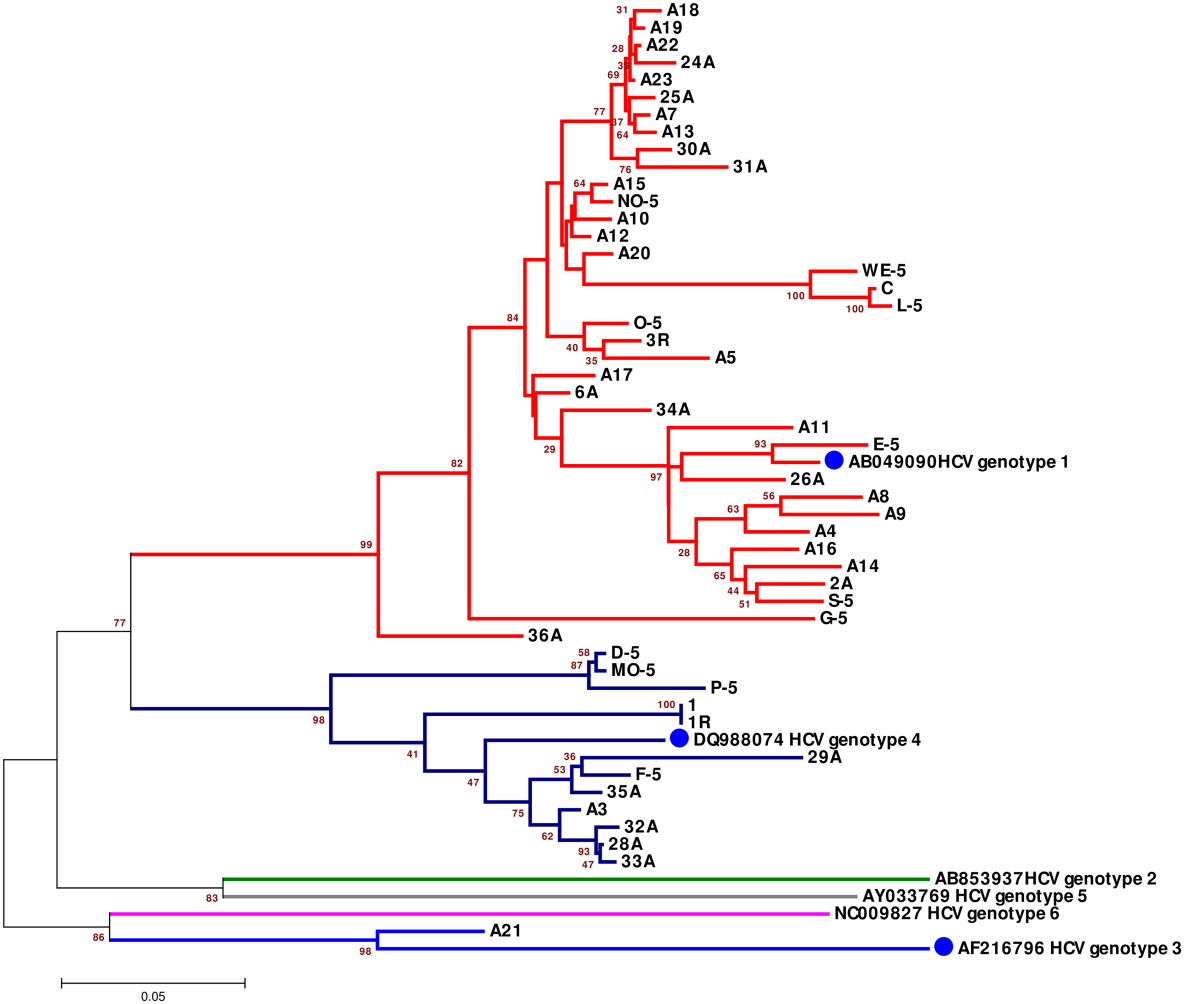
Phylogenetic tree based on six ~400-bp partial NS5B reference sequences representing all HCV genotypes (1–6). The phylogenetic tree was constructed using the neighbor-joining method (MEGA 6 software), and the reference sequences were retrieved from the DDBJ/EMBL/GenBank databases. All the reference isolates are indicated with their accession numbers and with closed blue circles. In addition, the 49 isolates from Saudi Arabia patients in this study whose nucleotide sequences were determined in the present study are shown. Thirty-six of the isolates clustered with HCV/1 and are represented by red branches, 12 isolates were grouped as HCV/4 and are represented by dark blue branches, and one isolate was grouped as HCV/3 and is represented by light blue branches. Bootstrap values indicate the major nodes as a percentage of the data obtained from 1000 replications.

The phylogenetic tree based on the ~400-bp *NS5B* partial gene from each sample and genes from the six reference HCV strains revealed three distinct clusters comparable to the six HCV genotypes. Thirty-six of the 49 (73.47%) isolates (2A, A4, A5, 6A, A7, A8, A9, A10, A11, A12, A13, A14, A15, A16, A17, A18, A19, A20, A22, A23, 24A, 25A, 26A, 30A, 31A, 34A, 36A, No-5, WE-5, C, L-5, O-5, 3R, E-5, G-5, and S-5) seemed to be more closely related to HCV genotype 1 based on nucleotide distance identity (mean: ±0.112), and HCV genotype 4 was verified in 12 (24.49%) isolates (D-5, Mo-5, P-5, 1, 1R, 29A, F-5, 35A, A3, 32A, 28A, and 33A); 1 (2.04%) isolate (A21) seemed to be related to HCV genotype 3 (Fig 3 and S4 Table), and the nucleotide distance (means: ±0.112 and ±0.179) verified this relationship. No samples were identified as HCV genotypes 2, 5, or 6 (Fig 3 and S4 Table).

### Identification of HCV subgenotypes based on the partial nucleotide sequence of the NS5B gene

**Fig 4:**
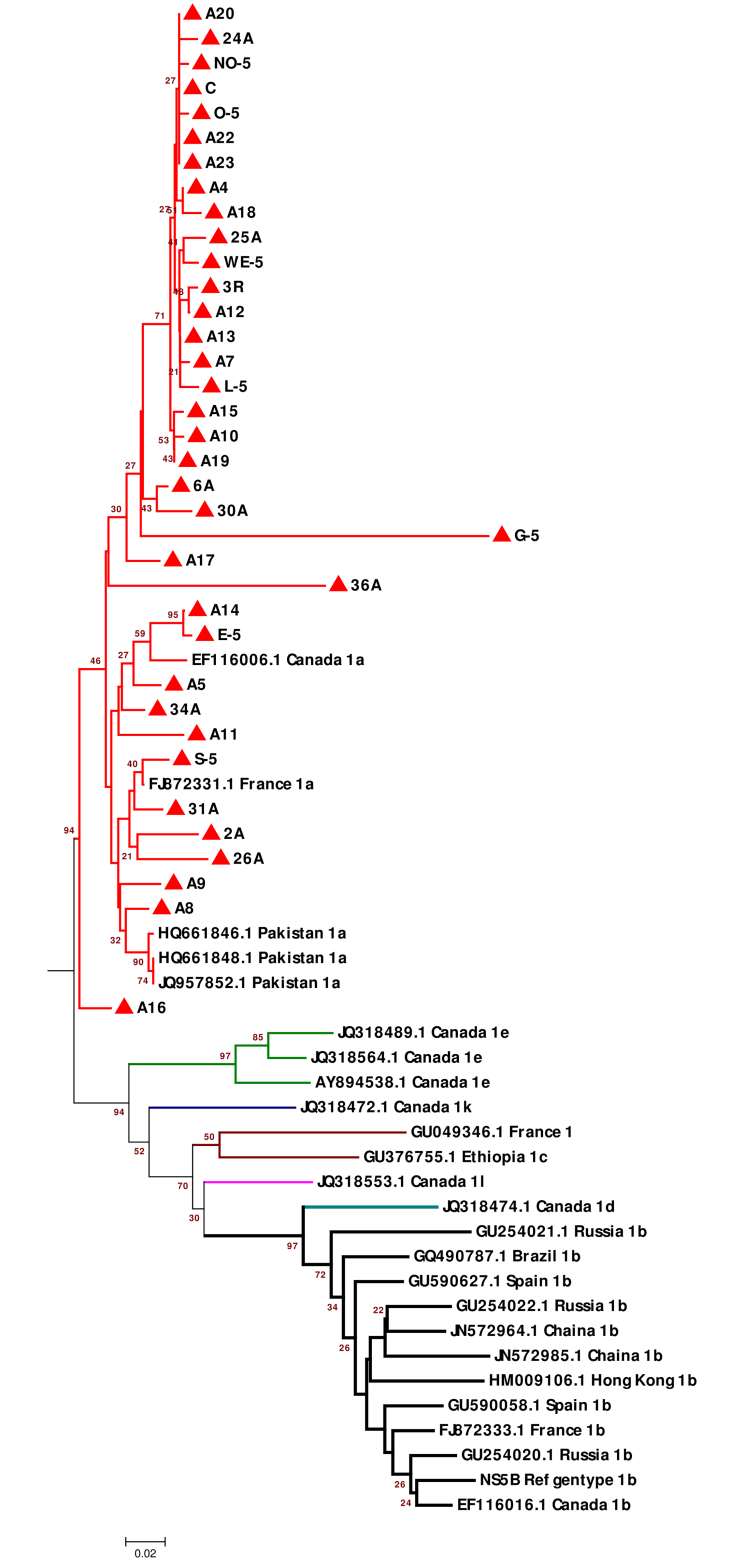
Phylogenetic tree for HCV/1 NS5B gene sequences constructed using the neighbor-joining method (MEGA 6 Software). Tree shows the phylogenetic relationship of 25 HCV-NS5B reference sequences and 36 HCV-NS5B sequences from this study. The reference HCV/1 subgenotype isolates are indicated by different colored branches along with their accession numbers and countries of origin. The 36 isolates from the present study are indicated by closed red triangles. Bootstrap values indicate the major nodes as a percentage of the data obtained from 1000 replications.

Partial *NS5B* reference sequences of 25 HCV/1 subgenotype strains, including the 36 isolates from the present study, were used to generate a phylogenetic tree. The reference sequences were retrieved from the DDBJ/EMBL/GenBank databases along with their accession numbers and country of origin for identification. All the 25 isolates were grouped into clusters that represented the seven subgenotypes of HCV/1 (1a, 1b, 1c, 1d, 1e, 1l, and 1k). An exclusive subset of the 36 HCV/1 isolates (2A, A4, A5, 6A, A7, A8, A9, A10, A11, A12, A13, A14, A15, A16, A17, A18, A19, A20, A22, A23, 24A, 25A, 26A, 30A, 31A, 34A, 36A, No-5, WE-5, C, L-5, O-5, 3R, E-5, G-5, and S-5) (100%) was observed to be closest to the reported HCV/1a strains, which included HCV/1a reference strains isolated from Pakistan, France, and Canada (Fig 4).

**Fig 5:**
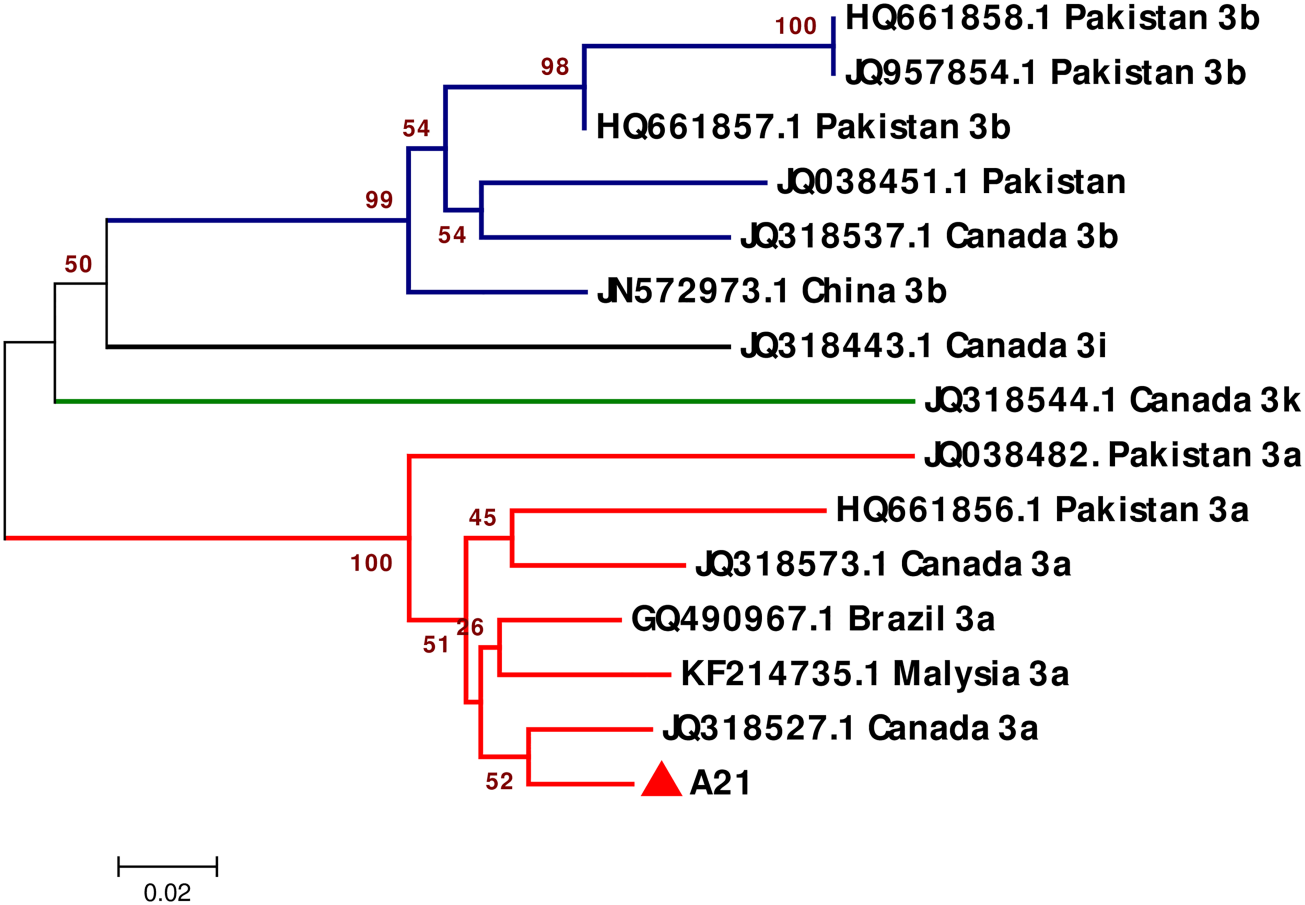
Phylogenetic tree of HCV/3 NS5B gene sequences constructed using the neighbor-joining method (MEGA 6 Software). Tree shows the phylogenetic relationship of 14 HCV-NS5B reference sequences and 1 HCV-NS5B sequence from the present study. Reference HCV/3 subgenotype isolates are indicated by different colored branches along with their accession numbers and countries of origin. A closed red triangle represents the isolate from the present study. Bootstrap values indicate the major nodes as a percentage of the data obtained from 1000 replications.

Another subgenotype phylogenetic tree was constructed for the 14 NS5B sequences of HCV/3 subgenotype strains retrieved from the DDBJ/EMBL/GenBank databases and the isolate from the present study. The isolate A21 from the present study was grouped with branches and seemed to be more closely related to the HCV/3a subgenotype. This identification was verified using HCV/3a reference strains from different countries such as Pakistan, Brazil, Malaysia, and Canada (Fig 5).

**Fig 6:**
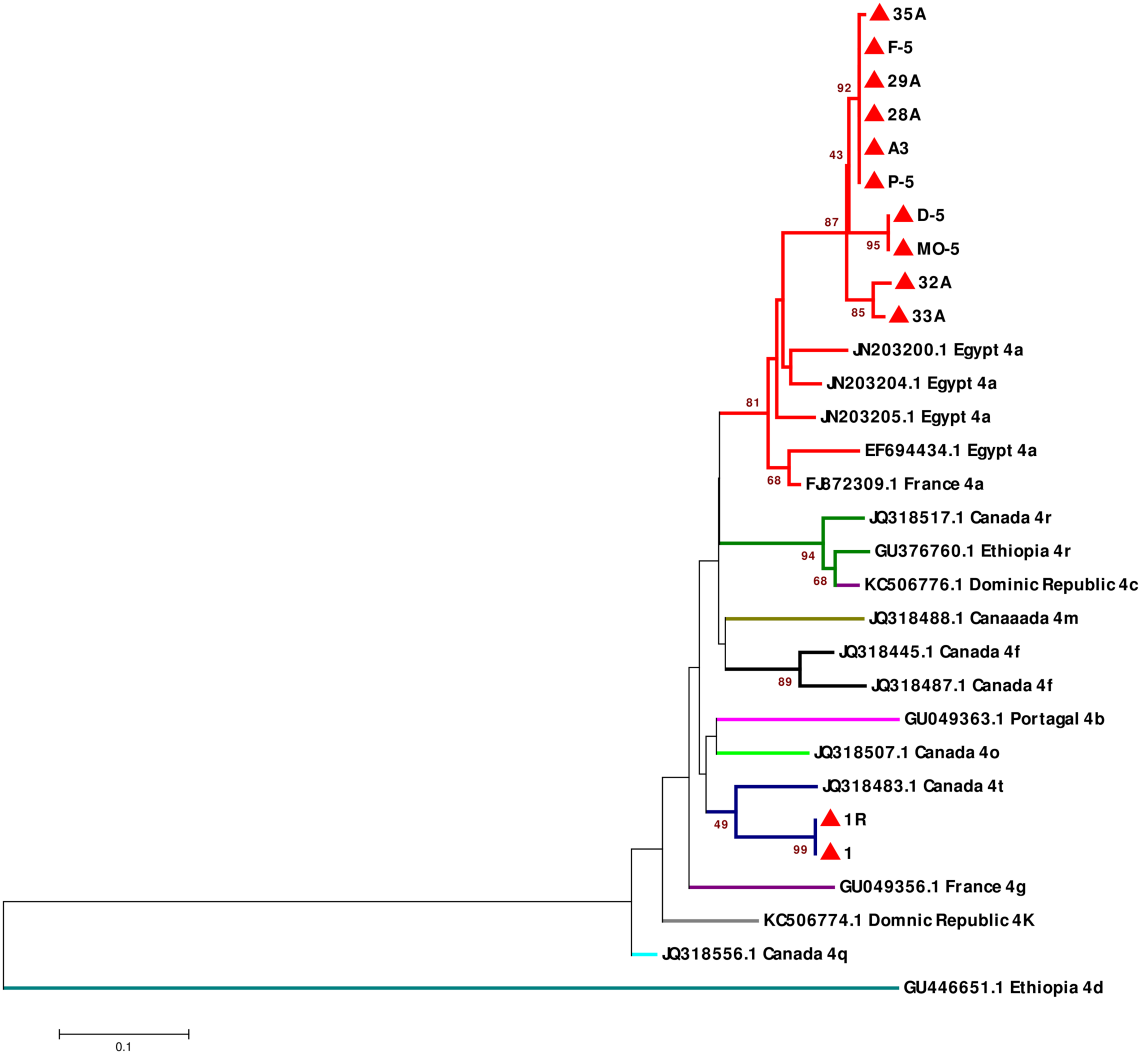
Phylogenetic tree of HCV/4 NS5B gene sequences constructed using the neighbor-joining method (MEGA 6 Software). Tree shows the phylogenetic relationship of 18 HCV-NS5B reference sequences and 12 HCV-NS5B sequences from the present study. Reference HCV/4 subgenotype isolates are indicated by different colored branches along with their accession numbers and countries of origin. The 12 isolates from the present study are indicated by closed red triangles. Bootstrap values indicate the major nodes as a percentage of the data obtained from 1000 replications.

A specific phylogenetic tree of the 12 remaining isolates corresponding to the HCV/4 genotype revealed that 10 (83.33%) of these 12 isolates (D-5, MO-5, P-5, 29A, F-5, 35A, A3, 32A, 28A and 33A) were related to the 4a subgenotype, and this result was verified using HCV/4a references strains from Egypt and France. The other two (16.67%) isolates (1R and 1) were defined as the HCV/4t subgenotype, but they assembled with reference strains isolated from Canada (Fig 6).

## Discussion

HCV has drawn attention for the unique geographic distribution of its six characteristic genotypes. Genotyping of HCV is routinely performed in many laboratories for providing counseling regarding treatment, classification, and epidemiology to monitor the distribution of virus strains and to identify risk factors for transmission and investigate contamination between individuals. Accurate subtype determination is necessary for epidemiology and risk factor of transmission between individual. [41–43]. In addition, the study of viral diversity provides a better understanding of the origins and dynamics of viral infections. Genetic variants of HCV are known to be widely spread around the world. Genotypes 1, 2, and 3 are found in all countries [44]. Comprehensive data on the distribution of HCV genotypes in Middle Eastern countries has recently been published [45], which indicates that HCV genotype 1 is largely predominant in some countries such as Turkey (82%), Cyprus (68%), and Iran (55%) [46–47], whereas HCV genotype 4 is predominant in Egypt, Saudi Arabia, Bahrain, and United Arab Emirates [24, 19, 25, 45, 48–52].

Analysis of the HCV genotype within a population is a useful epidemiological tool for study of the evolution of HCV infection in different geographical regions. HCV genotyping is also important because it provides information with regard to strain variations and potential association with disease severity [53]. Saudi Arabia has been classified as a country with an intermediate prevalence of HCV based on surveys conducted using samples from blood donors [54]. Despite the reported declines in HCV prevalence in the KSA over the last 10 years, HCV is still considered a major public health problem in the country, especially among hemodialysis patients and intravenous drug users [20, 55]. HCV has been found to be transmitted in the KSA through many routes including surgical operations, intravenous abuse [56], and sexual routes [57–58]. Currently, the characterization of HCV epidemiology in the KSA relies heavily on HCV seroprevalence studies, which are typically transversal studies and are conducted in select populations such as blood donors and hemodialysis patients [19–20]. Phylogenetic analysis of the 49 current isolates demonstrated that HCV genotype 1 is the most dominant HCV genotype (73.47%) among Saudi patients with chronic HCV using *HCV-NS5B* sequencing analysis. This result contrasts with previous observations that postulated that HCV genotype 4 appeared to be predominant in Middle Eastern countries, including Saudi Arabia; these reports were based on HCV-5'UTR sequencing analysis [48–52]

The choice of the genome region to be analyzed for identification of HCV genotypes and subgenotypes is crucial [59]. The *NS5B* region is highly informative in in phylogenetic analysis and has received the most attention for the characterization of HCV isolates worldwide [16, 27, 60–61]. With regard to *NS5B* nucleotide sequence genotypes, in the present phylogenetic investigation of the partial *HCV-NS5B* nucleotide sequence, we succeeded in genotyping 100% of the amplified samples. *HCV-NS5B* phylogenetic analysis revealed that the most prevalent genotypes were HCV/1 (73.47%), HCV/4 (24.49%), and HCV/3 (2.04%). Using pairwise distance analysis of the *NS5B* nucleotide sequence, we also succeeded in matching 100% of the current (amplified) isolates with their specific HCV genotypes using phylogenetic trees. Pairwise analysis of the isolates from the present study belonging to the HCV/1, HCV/4, and HCV/3 genotypes revealed average specific nucleotide distance identities of ±0.112, ±0.112, and ±0.179, respectively, without any interference between genotypes, which confirmed the outcome of our phylogenetic analyses. Many previous studies have indicated that the degree of accuracy of sequence variation of *NS5B* correlates well with HCV subtype definition, which relies on the highly informative character of specific *NS5B* motifs; in contrast, most classification errors caused by the use of the 5'NTR region are related to poor discriminating power and to the absence of target motifs specific for some subtypes. Therefore, we recommend that sequence analysis of the *NS5B* region be used rather than 5'NTR analysis for epidemiologic studies in the future [62–67].

In the current study, phylogenetic trees for different *NS5B* subtypes were constructed to estimate the precision of *NS5B-HCV* subtyping. The phylogenetic trees for the *HCV-NS5B* subtypes succeeded in classifying 100% of the HCV isolates. The common subtypes were 1a (73.47%), 4a (20.41%), 4t (4.08%), and 3a (2.04%). *NS5B* phylogenetic analysis of HCV/1 subtypes showed the grouping of all the strains in one branch, which confirmed that the common subtype of HCV genotype 1 among the isolates from the present study was 1a. This result contradicts that obtained using the 5'NTR phylogenetic trees generated in the present study as well as in other previous studies [19,25, 68]. One reason for this difference could be that the classification of isolates under the 1a subtype in the present study was mostly based on comparison with reference strains from Canada, Pakistan, and France.

Next, with regard to HCV/4 subtypes, the phylogenetic trees revealed that the common subtypes of HCV genotype 4 were 4a (83.33%) and 4t (16.67%). Although the strains from the current study defined as subtype 4a were generally similar to the reference strains isolated from France and Egypt, these strains defined as subtype 4a were clustered in a unique branch, which suggested that the patients in France and Egypt from whom the reference strains were isolated acquired their infection in their own country [69–70]. The isolates from the present study defined as subtype 4t were associated with reference strains from Canada.

With respect to the HCV/3 subtypes, although phylogenetic trees showed only one isolate from the present study as genotype 3, *HCV-NS5B* nucleotide sequence analysis successfully classified it as 3a subtype. The isolate from the present study with subtype 3a was found to be closely related to reference strains from Canada, Malaysia, and Pakistan.

HCV is a leading etiology of hepatocellular carcinoma (HCC). In Japan, Egypt, Saudi Arabia, southern Europe, and the USA, HCV is the main risk factor for HCC. Serological markers of HCV infection have thus been found in 71%, 80% 73%, and 39.5% of HCC cases from Egypt, Japan [71], the USA [72], and Saudi Arabia [73], respectively. These studies in agree with our data, which illustrated the most common HCV subtype is 1a (73.47%) and HCC developed in patients with either genotype 2a HCV or genotype 1b HCV [74]. Additionally, the current data will facilitate the clinical deployment of new protease inhibitors, which have significantly improved treatment outcome in patients with HCV genotype 1; HCV genotypes 1 and 4 were previously considered to be the most difficult to treat [75]

## Conclusion

To the best of our knowledge, this study is the first comprehensive research to address genotype and subgenotype analysis of HCV in the Jeddah province of Saudi Arabia using nucleotide sequencing. In this study, we aimed to determine the genotype and subgenotype for HCV isolates circulating in the Jeddah province through sequence analysis of the *NS5B* gene in a set of 49 HCV strains isolated from patients with chronic HCV infections from Saudi Arabia. Our results showed that the most common HCV genotypes circulating in Jeddah were genotypes 1, 4, and 3 (in that order), and some of the HCV isolates from the present study showed sequence similarity with some international HCV strains upon comparison with HCV reference sequences from Egypt, Morocco, Canada, Japan, India, and France.

Moreover, our study confirmed that *HCV-NS5B* gene sequence analysis provides precise genotype and subtype identification and an accurate epidemiological representation of circulating viral strains. The pairwise distance analysis also confirmed 100% of the results.

Although the molecular aspects of HCV infection were obtained through testing a small population in this study, the outcome confirmed the genotypes and subtypes of HCV that were circulating in Jeddah, Saudi Arabia. However, further studies with more samples need to be conducted to investigate if a shift in genotypes has occurred. Expanding the current study will allow us to follow changes in subtype distribution and to identify recombinants or even new subtypes or genotypes. Thorough insertion diagnostic methods that can assign viral genotype and subtype classifications are therefore greatly desired. Moreover, such methods will perform better when sequence variation from the NTR is eliminated and protein-coding regions such as NS5B are used instead. Indeed, further research that expands to cover all the regions of KSA is required to support the findings of the current study.

## Supporting information

S1 Table Characteristics of the 56 patients with chronic HCV infection enrolled in the present study. (DOCX)

S2 Table Complete genomes of HCV genotypes and NS5B reference sequences of subgenotypes retrieved from DDBJ/EMBL/GenBank databases. (DOCX)

S3 Table The current HCV samples codes and their accession numbers in the GenBank. (DOCX)

S4 Table Pairwise distances between the partial nucleotide sequence of the *NS5B* gene of HCV genotypes (1–6) and those of the 49 isolates from the present study generated using MEGA 6 software. (DOCX)
