# Supplementary material for: Partial sequencing analysis of the NS5B region confirmed the predominance of hepatitis C virus genotype 1 infection in Jeddah, Saudi Arabia
Source: PLoS One. 2017 May 26;12(5):e0178225. doi: 10.1371/journal.pone.0178225 (PMC5446157; doi:10.1371/journal.pone.0178225)
Supplement: S1 Table — (DOCX) [file pone.0178225.s001.docx]

**S1 Table. Characteristics of the 56 patients with chronic HCV infection enrolled in the present study.**

| Characteristic | | Number (%) | Mean | Standard Deviation | *P* value |
| --- | --- | --- | --- | --- | --- |
| Sex | Male | 23 (41) |  |  |  |
|  | Female | 33 (58) |  |  |  |
| Age (years) | <40 | 13 (23) |  |  |  |
|  | 40–59 | 33 (58.9) |  |  |  |
|  | >60 | 10 (17.85) |  |  |  |
| Nationality | Saudi | 47 (83.92) |  |  |  |
|  | Non-Saudi | 9 (16. 07) |  |  |  |
| ALT levels (U/L) | Normal | 41 (73.21) | 32.78 | + 11.92 | 0.0034* |
|  | elevated | 16 (28.57) | 101.94 | + 79.41 |  |
| AST levels (U/L) | Normal | 27 (48.21) | 25.77 | + 5.47 | 0.0004* |
|  | Elevated | 29 (51.72) | 83.83 | + 78.34 |  |
| AFP levels  (ng/mL) | Normal | 49 (87.5) | 4.864 | + 2.945 | 0.09 |
|  | Elevated | 7 (12.5) | 84.071 | + 103.9 |  |
| Viral Load (IU/mL) | Low ^(^**^1^**^)^ | 8 (14.2) | 23359 | +27580 | 0.031* |
|  | High ^(^**^2^**^)^ | 47 (83.92) | 11786302 | + 1489790 |  |

ALT, Alanine aminotransferase; AST, Aspartate Aminotransferase; AFP, Alpha-Fetoprotein; ^(^**^1^**^)^ Low viral load: ≤ 800000 IU/mL; ^(^**^2^**^)^ High viral load: >800000 IU/mL. *Significant increase at *P* < 0.05 as determined using the *t*-test.
