# Supplementary material for: Partial sequencing analysis of the NS5B region confirmed the predominance of hepatitis C virus genotype 1 infection in Jeddah, Saudi Arabia
Source: PLoS One. 2017 May 26;12(5):e0178225. doi: 10.1371/journal.pone.0178225 (PMC5446157; doi:10.1371/journal.pone.0178225)
Supplement: S2 Table — (DOCX) [file pone.0178225.s002.docx]

**S2 Table. Complete genomes of HCV genotypes and NS5B reference sequences of subgenotypes retrieved from DDBJ/EMBL/GenBank databases**.

| Reference sequences of the complete genome of HCV genotypes | Reference NS5B partial sequences of HCV subgenotypes | | |
| --- | --- | --- | --- |
|  | **Genotype 1 (Country of Origin)** | **Genotype 3 (Country of Origin)** | **Genotype 4 (Country of Origin)** |
| HCV/1 AB049090 | EF116006.1 (Canada) | HQ661858.1 (Pakistan) | JN203200.1 (Egypt) |
| HCV/ 2 AB853937 | FJ872331.1 (France) | JQ957854.1 (Pakistan) | JN203204.1 (Egypt) |
| HCV/ 3 AF216796 | HQ661846.1 (Pakistan) | HQ661857.1 (Pakistan) | JN203205.1 (Egypt) |
| HCV/4 DQ988074 | HQ661848.1 (Pakistan) | JQ038451.1 (Pakistan) | EF694434.1 (Egypt) |
| HCV/ 5 AY033769 | JQ957852.1 (Pakistan) | JQ318537.1 (Canada) | FJ872309.1 (France) |
| HC/ 6 NC009827 | JQ318489.1 (Canada) | JN572973.1 (China) | JQ318517.1 (Canada) |
|  | JQ318564.1 (Canada) | JQ318443.1 (Canada) | GU376760.1 (Ethiopia) |
|  | AY894538.1 (Canada) | JQ318544.1 (Canada) | KC506776.1 (Dominican Republic) |
|  | JQ318472.1 (Canada) | JQ038482 (Pakistan) | JQ318488.1 (Canada) |
|  | GU049346.1 (France) | HQ661856.1 (Pakistan) | JQ318445.1 (Canada) |
|  | GU376755.1 (Ethiopia) | JQ318573.1 (Canada) | JQ318487.1 (Canada) |
|  | JQ318553.1 (Canada) | GQ490967.1 (Brazil) | GU049363.1 (Portugal) |
|  | JQ318474.1 (Canada) | KF214735.1 (Malaysia) | JQ318507.1 (Canada) |
|  | GU254021.1 (Russia) | JQ318527.1 (Canada) | JQ318483.1 (Canada) |
|  | GQ490787.1 (Brazil) |  | GU049356.1 (France) |
|  | GU590627.1 (Spain) |  | KC506774.1 Dominican Republic |
|  | GU254022.1 (Russia) |  | JQ318556.1 (Canada) |
|  | JN572964.1 (China) |  | GU446651.1 (Ethiopia) |
|  | JN572985.1 (China) |  |  |
|  | HM009106.1 (Hong Kong) |  |  |
|  | GU590058.1 (Spain) |  |  |
|  | FJ872333.1 (France) |  |  |
|  | GU254020.1 (Russia) |  |  |
|  | EF116016.1 (Canada) |  |  |

All the reference sequences have been defined by their accession numbers and the country of origin
