# Supplementary material for: Partial sequencing analysis of the NS5B region confirmed the predominance of hepatitis C virus genotype 1 infection in Jeddah, Saudi Arabia
Source: PLoS One. 2017 May 26;12(5):e0178225. doi: 10.1371/journal.pone.0178225 (PMC5446157; doi:10.1371/journal.pone.0178225)
Supplement: S3 Table — (DOCX) [file pone.0178225.s003.docx]

**S3 Table: The current HCV samples codes and their Accession numbers in the GenBank**

| # | Sample code | Nucleotide Accession number | Protein Accession number |
| --- | --- | --- | --- |
| **HCV genotype 1** | | | |
|  | 2A | KX784096 | ARQ84056 |
|  | A5 | KX784099 | ARQ84059 |
|  | A7 | KX784101 | ARQ84061 |
|  | A12 | KX784106 | ARQ84066 |
|  | A14 | KX784108 | ARQ84068 |
|  | 24A | KX784117 | ARQ84077 |
|  | 25A | KX784118 | ARQ84078 |
|  | 26A | KX784119 | ARQ84079 |
|  | 31A | KX784121 | ARQ84081 |
|  | 36A | KX784123 | ARQ84083 |
|  | NO-5 | KX784126 | ARQ84086 |
|  | A23 | KX784116 | ARQ84076 |
|  | S-5 | KX784128 | ARQ84088 |
|  | 3R | KX784097 | ARQ84057 |
|  | L-5 | KX784125 | ARQ84085 |
|  | O-5 | KX784127 | ARQ84087 |
|  | WE-5 | KX784129 | ARQ84089 |
|  | E-5 | KX784124 | ARQ84084 |
|  | A10 | KX784104 | ARQ84064 |
|  | A13 | KX784107 | ARQ84067 |
|  | A8 | KX784102 | ARQ84062 |
|  | A11 | KX784105 | ARQ84065 |
|  | 30A | KX784120 | ARQ84080 |
|  | A22 | KX784115 | ARQ84075 |
|  | 34A | KX784122 | ARQ84082 |
|  | A19 | KX784113 | ARQ84073 |
|  | A17 | KX784111 | ARQ84071 |
|  | A16 | KX784110 | ARQ84070 |
|  | A15 | KX784109 | ARQ84069 |
|  | 6A | KX784100 | ARQ84060 |
|  | A9 | KX784103 | ARQ84063 |
|  | A4 | KX784098 | ARQ84058 |
|  | A18 | KX784112 | ARQ84072 |
|  | A20 | KX784114 | ARQ84074 |
|  | C | The sequence was submitted to GenBank and waiting for the Accession number. | |
|  | G-5 | The sequence was submitted to GenBank and waiting for the Accession number. | |
| **HCV genotype 4** | | | |
|  | 35A | KX810082 | AQS23729 |
|  | 28A | KX810078 | AQS23725 |
|  | 32A | KX810080 | AQS23727 |
|  | F-5 | KX810084 | AQS23731 |
|  | 33A | KX810081 | AQS23728 |
|  | P-5 | KX810086 | AQS23733 |
|  | MO-5 | KX810085 | AQS23732 |
|  | 29A | KX810079 | AQS23726 |
|  | D-5 | KX810083 | AQS23730 |
|  | A3 | KX810077 | AQS23724 |
|  | 1 | KX810071 | AQS23723 |
|  | 1R | KX810070 | AQS23722 |
| **HCV genotype 3** | | | |
|  | A21 | KX774463 | ARQ84090 |

Sequence of sample C

ATACCCGCTGTTTTGACTCCACAGTCACTGAGAGCGACATCCGTACGGAGGAGGCATCTACCAATGTTGTGACCTGGACCCCCAAGCCCGCGTGGCCATCAAGTCCCTCACCGAGAGACTTTATGTTGGGGGTCCTCTTACCAATTCAAGGGGGGAGAACTGTGGCTACCGCAGATGCCGCGCAAGCGGTGTGCTGACGACTAGCTGCGGTAACACCCTCACCTGCTACATCAAGGCCCGAGCAGCCTGTCGAGCCGCAGGGCTCCGGGACTGCACCATGCTCGTGTGTGGCGACGACTTAGTCGTTATCTGTGAAAGTGCGGGAGTCCAGGAGGACGCGGCGAACCTGAGAGCCTTCACGGAGGTATGACCAGGTATTCTGCTCCACCCGGAGATGCTCCACAGGCCACTTACGACCTTGAGCTTATCACATCTTGCTCCTCCAACGTCTCCGTGGCACGGGACGACAAGGGG

Sequence of sample G-5

CGCCTGCTTTGACTCTACAGTCACTGAGAGTGATATCCGTACGGAGGAGGCAATCTACCAATGTTGTGACCTGGACCCCCAAGCCCGCGTGGCCATCAGGTCCCTCACTGAGAGGCTTTACGTTGGGGGCCCTCTTACCAATTCAAGGGGGGAGAACTGCGGCTACCGCAGGTGCCGCGCGAGCGGCGTACTGACAACTAGCTGCGGTAACACCCTCACTTGCTACATCAAGGCCCAAGCAGCCTGTCGAGCCGCAGGGCTCCAGGACTGCACCATGCTCGTGTGTGGCGACGACCTAGTCGTTATCTGTGAAAGCGGGAACCCAGGAGGATGCGGCGAACCTACGAGTCTTCACGGAGGCTATGACTAGGTACTCTGCCCCCCCCGGGGACCCGCCCCAACCAGAATACGACTTGGAGCTTATAACATCATGTTCATCCAACGTCTCAGTCGCGCACGACGTGACGGGT
