# Supplementary material for: Partial sequencing analysis of the NS5B region confirmed the predominance of hepatitis C virus genotype 1 infection in Jeddah, Saudi Arabia
Source: PLoS One. 2017 May 26;12(5):e0178225. doi: 10.1371/journal.pone.0178225 (PMC5446157; doi:10.1371/journal.pone.0178225)
Supplement: S4 Table — (DOCX) [file pone.0178225.s004.docx]

**S4 Table.** **Pairwise distances between the partial nucleotide sequence of the *NS5B* gene of HCV genotypes (1-6) and those of the 49 isolates from the present study generated using MEGA 6 software.**

| **HCV 6** | **HCV 5** | **HCV 4** | **HCV 3** | **HCV 2** | **HCV 1** | **HCV samples** |
| --- | --- | --- | --- | --- | --- | --- |
| 0.373 | 0.377 | 0.114 | 0.389 | 0.422 | 0.352 | **1** |
| 0.373 | 0.377 | 0.114 | 0.389 | 0.422 | 0.352 | **1R** |
| 0.414 | 0.36 | 0.239 | 0.433 | 0.406 | 0.113 | **3R** |
| 0.407 | 0.399 | 0.354 | 0.454 | 0.413 | 0.092 | **2A** |
| 0.394 | 0.397 | 0.064 | 0.404 | 0.44 | 0.346 | **A3** |
| 0.434 | 0.376 | 0.366 | 0.486 | 0.369 | 0.093 | **A4** |
| 0.399 | 0.368 | 0.242 | 0.416 | 0.409 | 0.096 | **A5** |
| 0.411 | 0.38 | 0.282 | 0.454 | 0.392 | 0.076 | **6A** |
| 0.412 | 0.404 | 0.259 | 0.462 | 0.429 | 0.127 | **A7** |
| 0.441 | 0.381 | 0.389 | 0.496 | 0.365 | 0.053 | **A8** |
| 0.428 | 0.38 | 0.373 | 0.48 | 0.389 | 0.079 | **A9** |
| 0.397 | 0.396 | 0.275 | 0.462 | 0.412 | 0.108 | **A10** |
| 0.429 | 0.386 | 0.335 | 0.445 | 0.381 | 0.074 | **A11** |
| 0.395 | 0.384 | 0.271 | 0.454 | 0.405 | 0.097 | **A12** |
| 0.408 | 0.407 | 0.262 | 0.466 | 0.425 | 0.13 | **A13** |
| 0.432 | 0.395 | 0.34 | 0.446 | 0.409 | 0.086 | **A14** |
| 0.398 | 0.392 | 0.271 | 0.44 | 0.4 | 0.095 | **A15** |
| 0.421 | 0.397 | 0.335 | 0.456 | 0.386 | 0.087 | **A16** |
| 0.391 | 0.38 | 0.268 | 0.432 | 0.4 | 0.089 | **A17** |
| 0.398 | 0.408 | 0.252 | 0.454 | 0.433 | 0.13 | **A18** |
| 0.411 | 0.404 | 0.252 | 0.453 | 0.42 | 0.119 | **A19** |
| 0.399 | 0.373 | 0.282 | 0.441 | 0.393 | 0.087 | **A20** |
| 0.342 | 0.417 | 0.236 | 0.179 | 0.449 | 0.376 | **A21** |
| 0.411 | 0.399 | 0.252 | 0.444 | 0.416 | 0.122 | **A22** |
| 0.407 | 0.395 | 0.248 | 0.449 | 0.42 | 0.124 | **A23** |
| 0.428 | 0.42 | 0.262 | 0.465 | 0.428 | 0.13 | **24A** |
| 0.408 | 0.391 | 0.248 | 0.458 | 0.425 | 0.13 | **25A** |
| 0.406 | 0.402 | 0.339 | 0.441 | 0.382 | 0.063 | **26A** |
| 0.41 | 0.409 | 0.079 | 0.411 | 0.456 | 0.352 | **28A** |
| 0.411 | 0.404 | 0.262 | 0.45 | 0.416 | 0.113 | **30A** |
| 0.419 | 0.386 | 0.262 | 0.436 | 0.416 | 0.107 | **31A** |
| 0.427 | 0.421 | 0.079 | 0.411 | 0.456 | 0.353 | **32A** |
| 0.414 | 0.408 | 0.079 | 0.411 | 0.456 | 0.36 | **33A** |
| 0.402 | 0.388 | 0.282 | 0.44 | 0.409 | 0.091 | **34A** |
| 0.412 | 0.393 | 0.098 | 0.403 | 0.463 | 0.336 | **35A** |
| 0.401 | 0.39 | 0.199 | 0.432 | 0.416 | 0.159 | **36A** |
| 0.391 | 0.387 | 0.363 | 0.309 | 0.409 | 0.136 | **C** |
| 0.406 | 0.363 | 0.178 | 0.427 | 0.412 | 0.207 | **D-5** |
| 0.397 | 0.36 | 0.358 | 0.413 | 0.401 | 0.038 | **E-5** |
| 0.429 | 0.409 | 0.092 | 0.411 | 0.488 | 0.357 | **F-5** |
| 0.43 | 0.436 | 0.274 | 0.433 | 0.436 | 0.199 | **G-5** |
| 0.396 | 0.391 | 0.371 | 0.306 | 0.413 | 0.138 | **L-5** |
| 0.402 | 0.36 | 0.172 | 0.419 | 0.408 | 0.214 | **MO-5** |
| 0.407 | 0.393 | 0.268 | 0.45 | 0.409 | 0.1 | **NO-5** |
| 0.407 | 0.367 | 0.239 | 0.432 | 0.412 | 0.125 | **O-5** |
| 0.405 | 0.368 | 0.159 | 0.398 | 0.443 | 0.26 | **P-5** |
| 0.427 | 0.395 | 0.354 | 0.463 | 0.4 | 0.084 | **S-5** |
| 0.373 | 0.377 | 0.114 | 0.389 | 0.422 | 0.352 | **WE-5** |

Values represent the mean distances within each genotype and the isolates from the present study.

Brown cells represent the nearest distance between the isolate and the reference HCV genotypes, and this distance is considered as confirmation of the accurate categorization of the isolates under the different genotypes in the tree.
